# Expression of Recombinant Phosphodiesterases 3A and 3B Using Baculovirus Expression System

**DOI:** 10.15171/ijb.1400

**Published:** 2016-12

**Authors:** Yongmin Yan, Wenqian Jiang, Jingwen Liu, Wenrong Xu, Hui Qian

**Affiliations:** Jiangsu Key Laboratory of Medical Science and Laboratory Medicine, School of Medicine, Jiangsu University, Zhenjiang, Jiangsu, China

**Keywords:** Baculovirus Expression System, PDE3A, PDE3B, SF9 cells

## Abstract

**Background:**

Phosphodiesterase 3A (PDE3A) and phosphodiesterase 3B (PDE3B) play a critical role in the regulation of intracellular level of adenosine 3´,5´-cyclic monophosphate (cyclic AMP, cAMP) and guanosine 3´,5´-cyclic monophosphate (cyclic GMP, cGMP). Subsequently PDE3 inhibitors have shown to relax vascular and inhibit platelet aggregation in cardiovascular disease.

**Objectives:**

In this study, our aim was to establish a method of expression for the recombinant human PDE3A and PDE3B proteins in insect cells using baculovirus expression system in order to investigate the activity of the expressed PDE3A and PDE3B proteins.

**Materials and Methods:**

The full length human PDE3A and PDE3B cDNA were cloned into recombinant baculovirus and transfected into the SF9 insect cells. Recombinant proteins were collected at 48 h, 60 h, 72 h, and 84 h post transfection. Transfection of recombinant baculovirus was verified by the morphological changes of the SF9 cells. Expression of human PDE3A and PDE3B was detected by using RT-PCR and western blot, respectively. The ^125^I RIA method was used to determine the level of adenosine 3´,5´-cyclic monophosphate cAMP and cGMP, correspondingly. The activity of the expressed PDE3A and PDE3B proteins were investigated by cAMP and cGMP dsgradation with or without addition of milrinone, a potent and selective PDE inhibitor.

**Results:**

Recombinant human PDE3A and PDE3B proteins were stably expressed in SF9 cells and could be detected by distinct morphological changes in the SF9 cells, RT-PCR, and western blot at 48 h post-transfection. The molecular weights of the recombinant PDE3A and PDE3B molecular weights proteins were about 120 KDa and 135 KDa, respectively. Results of ^125^I RIA assay showed that the levels of cAMP and cGMP were significantly decreased after incubation with the expressed PDE3A and PDE3B proteins. Furthermore, degradation of cAMP and cGMP through the activity of PDE3A and PDE3B was suppressed following to the addition of milrinone.

**Conclusions:**

Recombinant human PDE3A and PDE3B could be expressed in SF9 cells using baculovirus expression system, and thus provides the basic material for studying human PDE3A and PDE3B activity.

## 1. Background

Phosphodiesterases (PDE) are a diverse family of enzymes that significantly regulate cyclic nucleotides hydrolysis and effect on cell functions. Both PDE3A and PDE3B can hydrolyze cyclic adenosine monophosphate (cAMP) and cyclic guanosine monophosphate (cGMP), as well as being involved in the regulation of cardiovascular functions, insulin secretion, and the lipid metabolism (1-3). Selective PDE3 inhibitors have antithrombotic, antiproliferative, bronchodilatory, anti-inflammatory, and positive inotropic effects (4,5). Several PDE3 inhibitors, including cilostamide and milirinone have been used for heart failure therapy. However, clinical application of these inhibitors was limited by a number of side effects (4,6). It is an attractive subject to produce active human PDE3 for the screening of new inhibitors with the desired pharmacological properties and lower side effects. Native PDE3A has been isolated from platelets and human erythroleukemia cells (7). Recombinant PDEs also have been expressed in *Escherichia coli* (8). But, expression of human PDE3 in eukaryotic expression system has not been investigated as yet. Thousands of eukaryotic proteins including proteins that are used for human vaccines production have been expressed in the insect cells using Baculovirus Expression System (BES) (9). Compared to the mammalian cells, BES facilitates expression of eukaryotic protein more rapidly and efficiently without the need for extra facilities.

## 2. Objectives

In this study, we used a developed baculovirus expression ready system and the human PDE3A and PDE3B cDNAs have been cloned into the recombinant baculovirus. We aimed to use Baculovirus expression system to express human PDE3A and PDE3B for PDE3 inhibitor selection.

## 3. Materials and Methods

### 3.1. Materials

SF9 insect cells were obtained from the School of Life Science, Jiangsu University; SF900-II (Invitrogen, CA, USA); baculo-EZTM Expression Ready Full Length Collection- human PDE3A and PDE3B were purchased from (Orbigen, USA), total RNA Isolation Kit and SuperScript™ One-Step RT-PCR Systems were purchased from (Invitrogen, CA, USA), primary antibodies for PDE3 and HRP-conjugated IgG (Santa Cruz, CA, USA), Milrinone was obtained from Huzhou Hengyuan Biological limited company, Taq DNA polymerase was purchased from (TaKaRa, China), ^125^I RIA cGMP, and ^125^I RIA cAMP Kits were obtained from (Shanghai University of Traditional Chinese Medicine).

### 3.2. Transfection of Recombinant PDE3A and PDE3B Baculovirus in SF9 Cells

SF9 cells were maintained in Grace’s insect cell culture medium (10% FBS) and adapted to the culture condition of Sf-900 II medium at 27°C. For the baculovirus transfection, SF9 cells were seeded into a 25 cm^2^ flasks at 5×10^5^ cells.mL^-1^ and transfected with recombinant baculovirus. The morphology of the transfected cells was visualized using an Olympus Fluorescence Microscope (magnification 200X). At 48-96 h post infection, transfected cells were centrifuged at 500 Xg for 5 mins. The supernatant was stored at -70ºC for further investigation. After washing twice with PBS (0.1 mol.L^-1^, pH 7.4), cells were collected for RNA and protein assays.

### 3.3. Reverse-Transcription PCR

Evaluation of PDE3A and PDE3B mRNA levels was conducted by RT-PCR. 1 μg of the total RNA was used for reverse transcription of RNA into cDNA using SuperScript™ One-Step RT-PCR Systems according to the manufacturer’s protocol. The primers that were designed for PCR analysis are shown in Table 1.

**Table 1:** Primers for RT-PCR

** Genes**	** Primer Sequences (5'-3')**	** Annealing Temperature(°C) **	** Product size (bp) **
PDE3A PDE3B	For: CGCTGCTCTTCGTC TCC Rev: ACGCCAGGTAAGGTCTCC For: CGTTCTTCTCCTCAACTAGC Rev: TTCCTCTTCATCTGCCTCTT	63 58	310 400

### 3.4 . Western Blot Assay

Collected SF9 cells were lysed with HEPES lysis buffer (pH 7.4, 20 mM HEPES, 1 mM DTT, 1 mM EDTA, 100 mM PMSF, 10 mg.mL^-1^ leupeptin). The concentration of the collected cellular protein following to the cell lysis was detected using Bradford method. 10-30 μg of protein was separated on 10% SDS-PAGE and transferred onto PVDF (polyvinylidene difluoride) membrane. For analysis of PDE3 expression percentage, Coomassie brilliant blue stained PAGE gel was scanned and analyzed using Synaeme Bioimaging System. For western blot assay, the transferred membrane was incubated with anti-PDE3A (1:1000), anti-PDE3B (1:500), and HRPlinked anti-mouse IgG sequentially. Immunoreactive proteins were detected with Amersham’s enhanced chemiluminescence system.

### 3.5. PDE3 Activity Assay

PDE3 activity was determined by enzymatic cGMP and cAMP hydrolysis. Different doses of expressed PDE3A and PDE3B proteins were added and incubated with 2 μM cAMP or cGMP at 30°C for 10 min respectively and the reactions were stopped with boiling water for 1 min. Then the un-hydrolyzed cGMP and cAMP were detected using ^125^I RIA cGMP and ^125^I RIA cAMP Kit according to the manufacturer’s protocol. Briefly, the labeled ^125^I-cAMP/^125^I-cGMP was precipitated with anti-cAMP/anti-cGMP. Radioactivity of unreacted ^125^IcAMP/^125^I-cGMP in the supernatant was detected by γ radioimmunity counter. The concentration of cAMP/cGMP was calculated using Line Weaver-Burk plots with standards (concentrations from 0.04 to 20 μM). To analyze the inhibition of PDE3A and PDE3B activity, milrinone was added and incubated with PDE3 and cGMP or cAMP. The concentration of cGMP or cAMP was detected as described above. IC50 values of the PDE3 on cGMP and cAMP was calculated by LOGIT method.

### 3.6. Statistical Analysis

Data were expressed as mean ± standard deviation (SD). Significance was analyzed with Student’s t-test (2-tailed) and P<0.05 was considered as significant.

## 4. Results

### 4.1. Recombinant PDE3A and PDE3B Transfection; SF9 cells’ Morphological Changes at Different Periods

**Figure 1:**
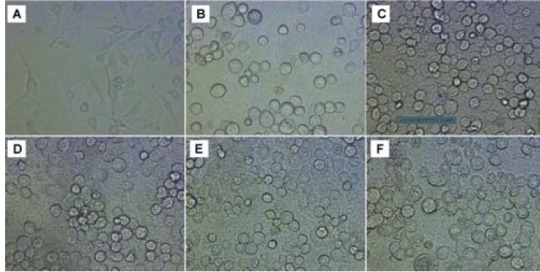
The morphological changes of the SF9 cells after transfection with the recombinant PDE3A and PDE3B. A: Control (non-transfected), B: SF9 cells were transfected with AcPAK6, C: recombinant baculovirus containing human PDE3A gene at 48 h D: 72 h, E: PDE3B gene at 48 h, F: 72 h. The original magnification was set at 200×. Abbreviations: PDE3A: phosphodiesterase 3A, PDE3B: phosphodiesterase 3B, AcPAK6: control baculovirus without insertion of PDE gene

The morphology of the SF9 cells observed before and after recombinant baculovirus transfection. In comparison to the non-transfected control SF9 cells (Figure 1A), cells stopped proliferation and production of the budded virus were increased at 6 to 24 h in AcPAK6 control (Figure 1B), PDE3A (Figure 1C, D) and PDE3B (Figure 1E, F) baculovirus group. Transfected cells were observed with an increased diameter and enlarged nuclei under phase contrast microscopy (Figure 1B, F). At 84 h post infection, cell started to float and produced the non-occluded virus. These results have indicated that SF9 cells were successfully transfected with baculovirus.

### 4.2. PDE3A,3B Expression Evaluation at mRNA and Protein Levels

**Figure 2:**
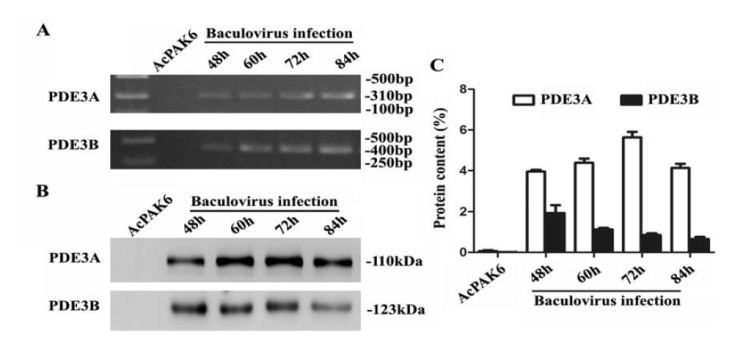
Expression of the PDE3A and PDE3B in the infected SF9 cells as detected by RT-PCR and western blot. A: Following to the transfection with the recombinant human PDE3A and PDE3B virus, expressions of PDE3A and PDE3B mRNA were detected at 48 h, 60 h, 72 h and 84 h in SF9 cells post-transfection by RT-PCR. B: Expression of human PDE3A and 3B proteins was detected using western blotting. C: The percentage of the expressed human PDE3A and PDE3B proteins was estimated in the total protein according to the measured protein concentration. PDE3A: phosphodiesterase 3A, PDE3B: phosphodiesterase 3B, AcPAK6: control baculovirus without an inserted PDE gene.

Next, we investigated the expression of the PDE3A and PDE3B in the transfected SF9 cells with the recombinant baculovirus. To determine human PDE3A and PDE3B mRNA in the infected SF9 cells, RT-PCR was used. Results showed the amplified RTPCR products of 310 bp and 400 bp pairs for PDE3A and PDE3B mRNA, respectively. These results suggested that transcript levels of PDE3A and PDE3B were detected in the infected cells (Figure 2A).

To examine the expression profile, PDE3A and PDE3B protein were separated using SDS-PAGE and were detected applying western blotting. Result showed that human PDE3A and PDE3B could be detected at 48 h, 60 h, 72 h, and 84 h post transfection compared to the AcPAK6 baculovirus transfection (Figure 1B). The percentages of the expressed PDE3A to the total protein were 4.2%, 4.5%, 5.8%, and 4.3% at different periods (Figure 2c). For the PDE3B expression, the percentages were 2.1%, 1.3%, 0.9% and 0.5% (Figure 2c). These results suggested that PDE3A and PDE3B expression in SF9 cells reach a peak at 72 h and 48 h, respectively.

### 4.3. Effect of Recombinant PDE3A and PDE3B on cAMP/cGMP Hhydrolysis

**Figure 3:**
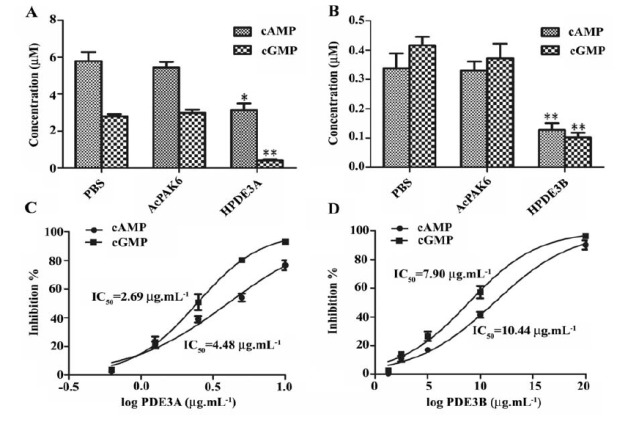
Effects of recombinant PDE3A and PDE3B on cyclic AMP and cyclic GMP hydrolysis. A: Cyclic AMP (cAMP) and cyclic GMP (cGMP) were degraded by the expressed recombinant human PDE3A protein compared to the controlled PBS and AcPAK6 protein. B: Cyclic AMP (cAMP) and cyclic GMP (cGMP) were degraded by the expressed recombinant PDE3B protein compared to the controlled PBS and AcPAK6 protein. C: The IC50 values for the PDE3A on cAMP and cGMP were 4.48 ìg.mL^-1^ and 2.69 ìg.mL^-1^. D: The IC50 values of PDE3B on cAMP and cGMP were 10.44 ìg.mL^-1^ and 7.9 ìg.mL^-1^. *, P<0.05, **, P<0.01, respectively. Abbreviations: PDE3A: phosphodiesterase 3A, PDE3B: phosphodiesterase 3B, AcPAK6: control baculovirus without an inserted PDE gene

**Figure 4:**
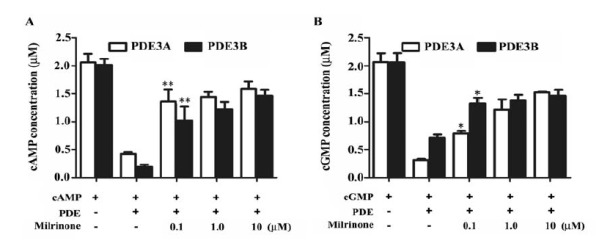
The inhibitory effect of the milrinone on the recombinant PDE3A as well as PDE3B induced cAMP and cGMP hydrolysis. A: The level of cyclic AMP (cAMP) was detected after incubation with the recombinant human PDE3A and PDE3A with or without selective PDE inhibitor, milrinone (0.1 μM, 1.0 μM, and 10 μM). B: The level of cyclic GMP (cGMP) was detected after incubation with recombinant human PDE3A and PDE3B with or without selective PDE inhibitor, milrinone (0.1 μM, 1.0 μM, 10 μM) addition. *, P<0.05, **, P<0.01. Abbreviations: PDE3A: phosphodiesterase 3A, PDE3B: phosphodiesterase 3B

PDE3 were described as enzymes that hydrolyze both cGMP and cAMP with a high affinity for the substrate (9). We evaluated the effects of the expressed human PDE3A and PDE3B on hydrolysis of the cAMP/cGMP. In comparison with PBS and AcPAK6 group, cAMP and cGMP were significantly degraded by the addition of PDE3A and PDE3B (Figure 3 A,B) (*, *P*<0.05, **, *P*<0.01). The IC50 values of PDE3A on cAMP and cGMP were 4.48 μg.mL^-1^ and 2.69 μg.mL- 1, respectively. The IC50 values of PDE3B on cAMP and cGMP were 10.44 μg.mL^-1^ and 7.9 μg.mL^-1^, as well. Milrinone is a potent and selective PDE3 inhibitor. Addition of milrinone dose-dependently reversed human PDE3A and PDE3B induced cAMP/cGMP hydrolysis (Figure 4A and 3B) (*, *P*<0.05, **, *P*<0.01). These results have indicated that the expressed human PDE3A and PDE3B proteins have the full enzymatic activity and could be inhibited by milrinone.

## 5. Discussion

Selective PDE3 inhibitors, including enoximone, cilostazol, milrinone, and OR-1896 have been used in cardiovascular, pulmonary, and the kidney disease therapy (10-12). For example, OR-1896 elicits a positive inotropic response (PIR) to levosimendan for treatment of the heart failure that is mediated primarily through PDE3 inhibition (13). Dietrichs *et al*. (14) have found that milrinone ameliorated cardiac dysfunction during rewarming from hypothermia by preventing cAMP breakdown and PDE3 activity. However, some drugs such as milrinone were reported to be associated with the risk of mortality (4,6,12). Nonetheless, they are still used in heart failure and intermittent claudication respectively (15-17). Thus, there still remains a challenge to achieve enough active PDEs as well as producing the better PDE inhibitors for clinical use.

Native PDE3A has been isolated from platelets and human erythroleukemia cells (7). Isolation of the native PDE3B has not been reported. However, the native PDE3A content in the platelets and the cells are very low and the isolated PDE3A is not sufficient enough for a large-scale inhibitor screening. Also, recombinant PDEs have been expressed in *E. coli* (8). The activity of prokaryotic protein expressed in *E. coli* is not the same as eukaryotic protein in human. In this study, human PDE3A and PDE3B cDNAs were cloned into the recombinant baculovirus. With this developed baculovirus system, we can express target protein in one week without the need for the extra facilities. Therefore, it is an ideal method to use BES for PDE3A and PDE3B production in SF9 cells.

Baculovirus infected SF9 cells will follow a lytic pathway. Harvesting before SF9 cells lysis will minimize the degradation of the expressed protein by cellular proteases released in the cell culture. To identify the infection of human PDE3A and PDE3B, we observed the altered morphologies of SF9 cells between 48 and 96 h post-infection. The cells were developed virogenic stroma under phase contrast microscopy at 48 h post baculovirus infection, which was consistent with the manual of BES. RT-PCR results have also shown the existence of PDE3 mRNA in the transfected cells. Because the reference gene can not be found in SF9 insect cells, we failed to use qRT-PCR to quantify the mRNA levels of human PDE3.

It has been reported that native human PDE3A and PDE3B has a molecular weight of 110 kDa and 123 kDa, respectively (18,19). Result of western blot analysis showed that PDE3A and PDE3B were successfully expressed and increased in the recombinant PDE3 baculovirus infected SF9 cells compared to AcPAK6 control cells. PDE3 activity has been identified by monitoring the reduction in the level of cAMP or cGMP (13,20). For assaying the activity of the recombinant proteins, PDE3 were incubated with cAMP and cGMP, respectively. As expected, recombinant PDE3A and PDE3B have caused a significant reduction in the level of cAMP and cGMP. However, a higher IC50 for cAMP and cGMP was found for the PDE3A and PDE3B recombinants from SF9 insect cells compared to the corresponding recombinant proteins from *E. coli* strain TOP 10 (8), suggesting that a purified PDE3 should be used for future analysis. Milrinone is a selective PDE3 inhibitor that is used as a drug which can inhibit the activity of PDE3 (11,14,17). To further demonstrate the activity of PDE3, milrinone was used to reverse the hydrolytic effect of PDE3 on cAMP and cGMP. Addition of milrinone has reduced PDE3 activity dose-dependently and resulted in an increased cAMP/cGMP levels, which was similar to those function of milrinone in cardiac function and heart failure (15,21). Our results indicate that PDE3A and PDE3B that were expressed in the SF9 cells have full enzymatic activity and could be used for PDE inhibitors selection.

In conclusion, our study has established an approach for expressing human PDE3A and PDE3B in SF9 cells using baculovirus expression system. As well, it provides the basic material for studying its bioactivity and screening of the selective PDE3A and PDE3B inhibitors. Our study has also demonstrated that we can obtain enough PDE3A and PDE3B for the research purposes with this method.

## Acknowledgements

This work was funded by the National Natural Science Foundation of China (Grant no. 81200312, 81670549, 81670502), China Postdoctoral Science Foundation (2015M580403, 2016T90431), Natural Science Foundation of Jiangsu Province (Grant no. BE2016717, BK2012288), the Scientific Research Foundation of Jiangsu University (grant no. 11JDG062), Priority Academic Program Development of Jiangsu Higher Education Institutions (PAPD), Young backbone teacher training project of Jiangsu University.
